# Synergy Between Direct Coronary Stenting Technique and Use of the Novel Thin Strut Cobalt Chromium Skylor™ Stent: the Mace in Follow Up Patients Treated with Skylor Stent [MILES Study]

**DOI:** 10.2174/157340312801215818

**Published:** 2012-02

**Authors:** Arturo Giordano, Michele Polimeno, Nicola Corcione, Luciano Fattore, Luigi Di Lorenzo, Giuseppe Biondi-Zoccai, Paolo Ferraro, Maria Fiammetta Romano

**Affiliations:** 1 Unità di Cardiologia Invasiva, Clinica Pineta Grande, Castelvolturno (CE), Italy; 2 Unità di Cardiologia Invasiva, Clinica S. Lucia, S. Giuseppe Vesuviano (NA), Italy; 3 U.O.C. Cardiologia - UTIC - P.O. S. Giuseppe e Melorio SMCV (CE), Italy; 4 U.O.C. Cardiologia – UTIC, P.O. S. Rocco, Sessa Aurunca (CE), Italy; 5 Dipartimento di Scienze e Tecnologie Medico-Chirurgiche, Sapienza Università di Roma, Latina, Italy; 6 Dipartimento di Biochimica e Biotecnologie Mediche, Università Federico II, Napoli, Italy

**Keywords:** Angioplasty, stents, restenosis.

## Abstract

**BACKGROUND:**

Despite significant improvements in stent platform, currently available bare-metal stents (BMS) are still associated with restenosis. Thin-strut design cobalt-chromium alloys hold the promise of improving results of BMS, especially when implanted with direct technique. We performed an observational study to appraise outcomes of the novel Skylor™ stent, stratifying outcomes according to stenting technique.

**METHODS and RESULTS:**

We included all consecutive patients undergoing coronary stenting with Skylor™ at 2 centers between 2006 and 2009. The primary end-point was the long-term rate of major adverse cardiac events (MACE, i.e. death, myocardial infarction (MI), coronary artery bypass grafting (CABG) or target vessel revascularization (TVR)). As pre-specified analysis, we compared patients undergoing direct stenting versus those stent implantation following pre-dilation. A total of 1020 patients were included (1292 Skylor™ stents), with procedural success obtained in 99%. Comparing patients undergoing direct stenting (66%) versus pre-dilation (34%) at 16±7 months of follow-up, MACE had occurred in, respectively, 8% versus 14% (p=0.001), with death in 1% versus 2% (p=0.380), MI in 1% versus 2% (p=0.032), CABG in 0.2% versus 2% (p=0.012), and TVR in 6% versus 9% [p=0.071]. Even at multivariable analysis with propensity adjustment, direct stenting was associated with significantly fewer MACE [hazard ratio 0.60 [0.38-0.93], p=0.024].

**CONCLUSIONS:**

This observational study suggests the presence of a beneficial synergy between direct coronary stenting technique and use of the novel thin-strut cobalt-chromium Skylor™ stent in real-world patients undergoing PCI.

## INTRODUCTION

Despite ongoing evidence for the benefits of medical therapy in low or moderate risk patients [1-2], and coronary artery bypass grafting (CABG) in high-risk patients [2-3], percutaneous coronary intervention (PCI) maintains an important clinical role in patients with stable coronary disease failing best medical therapy and those with unstable coronary disease [4].

Drug-eluting stents (DES) have been proved significantly superior to stainless-steel bare-metal stents (BMS) [5], but their premium efficacy in comparison to more sophisticated BMS has been questioned, especially in patients and lesions at lower risk of restenosis or higher risk of thrombosis [6-7]. Thus, in several countries BMS are still used in up to 50-60% of all PCI [8]. Yet, despite significant improvements in stent platform and alloys, currently available BMS are still associated with restenosis [5-6]. Thin-strut designs and cobalt-chromium alloys have been suggested to improve early and long-term outcomes of PCI with BMS by, respectively, reducing the risk of side branch occlusion leading to peri-procedural myocardial infarction (MI) and decreasing the incidence of restenosis leading to repeat revascularization [9-10]. This holds even truer when a direct stenting technique is employed, which minimizes geographical miss [11-12]. Specifically, promising preliminary results have been reported on the novel thin-strut designs cobalt-chromium Skylor™ (Medtronic-Invatec, Roncadelle, Italy) stent [10]. However, no thorough and comprehensive appraisal of this stent in real-world patients, including both those at high as well as low risk of adverse events, is available. We thus performed a retrospective observational study to appraise outcomes of the Skylor™ stent, stratifying outcomes according to stenting technique.

## METHODS

The Mace In foLlow up patiEnts treated with Skylor stent (MILES) study was a retrospective observational registry involving two high-volume PCI centers. All consecutive patients undergoing coronary stenting with Skylor™ between 2006 and 2009 were included, with the notable exception of lack of written informed consent. Thus, all patients provided written informed consent, and ethical approval was waived given the observational design of the study.

The decision to perform PCI with BMS instead of DES reflected the current practice and strategy of each center according to its indications and protocols, as well as the individual operator’s judgment. Similarly, direct stenting was at the operator’s discretion. However, agreed indications for BMS included lesions at low or moderate risk of restenosis, patients at high risk of thrombosis, bleeding or requiring non-cardiac surgery within 12 months, whereas typical contraindications included unprotected left main disease, in-stent restenosis, or diffuse diabetic coronary disease. Accordingly, agreed indications for direct stenting included thrombotic lesions, in particular acute MI, American College of Cardiology/American Heart Association type A or B1 lesions, saphenous bypass grafts, and ostial lesions. Conversely, direct stenting was generally contraindicated in highly calcified lesions, distal protected left main disease, or true bifurcation lesions. 

The use of all interventional techniques and devices [including stent size, inflation pressure, intravascular ultrasound guidance, and intra-aortic balloon pump), as well as the administration of therapies before, during the procedure or afterwards [including intravenous glycoprotein IIb/IIIa inhibitors, dual antiplatelet therapy and other medical treatments for coronary artery disease such as angiotensin-converting enzyme inhibitors, aldosterone receptor antagonists, beta-blockers, nitrates and statins) and access site was left to the cardiologist’s discretion. 

Creatin-phosphokinase muscle-brain isoenzyme levels were measured in all patients after 6-12 hours and again the morning after the intervention. Clinical follow-up was performed at 6 months or more through office visits, telephone interviews, or, when patients could not be contacted either way, by consulting civil registries of mortality.

The primary objective of the study was to appraise the risk-benefit balance of percutaneous coronary intervention (PCI) with the Skylor™ stent at 6-month follow-up. Secondary objective was the comparison of patients undergoing PCI with direct stenting versus those undergoing PCI with pre-dilation.

Thus, the primary end-point was the long-term rate of major adverse cardiac events (MACE, i.e. the composite of death, MI, CABG, or Skylor™ target vessel revascularization (TVR, i.e. revascularization in a vessel previously treated with a Skylor™ stent). Secondary end-points included individual components of MACE, target lesion revascularization (TLR), TVR, any repeat PCI, Academic Research Consortium definite stent thrombosis, stroke, and binary angiographic restenosis.

Categorical variables are expressed as n/N and %. Continuous variables are expressed as mean±standard deviation. Categorical variables were compared with Fisher and Pearson chi-squared tests, when appropriate. Continuous variables were compared with Gosset’s test. Bivariate survival analysis was performed with the Kaplan-Meier method, comparing survival curves with the log-rank test. Multivariable survival analysis was performed with Cox proportional hazard models to appraise the impact of direct stenting technique on the risk of adverse events, by building an enter model including all variables associated with at least borderline significance (p<0.10), as well as other traditionally relevant covariates [thus age, diabetes mellitus, hypercholesterolemia, renal failure, left ventricular ejection fraction (LVEF)<0.35, acute or recent myocardial infarction, multivessel disease, treated lesions, PCI of left main, type B2 or C lesion, total occlusion, bifurcation, calcification, tortuosity, long lesion, small vessel [reference diameter≤2.75 mm], stent diameter, stent length, and post-dilation]. In addition, a propensity score was fitted in the regression model to further adjust for residual confounders [13]. Results are reported as hazard ratios (HR), with 95% confidence intervals. All reported p values are 2-tailed and unadjusted for multiple comparisons. No mathematical transformations or imputations for missing data were performed, and all computations were performed with SPSS-PASW 18.0 (IBM, Armonk, NY, USA).

## RESULTS

A total of 1020 patients were included, undergoing implantation of 1292 Skylor™ stents, which were divided in those undergoing direct stenting (66%) versus pre-dilation (34%). A number of significant differences between these two groups were found in baseline (Tables **1**), angiographic and procedural features (Table **2**). Specifically, a direct stenting technique was more frequently performed in younger patients (p=0.001), and in those with fewer lesions (p=0.027), whereas pre-dilation was more frequent in those being treated in the left main (p=0.013), with tortuous vessels (p=0.009), small vessels (p<0.001), bifurcations (p=0.001), total occlusions (p<0.001), type B2 or C lesions [p<0.001], calcific lesions (p=0.008), and long lesions (p<0.001). Irrespective of the technique used, procedural success was above 98%, despite the unselected patient population.

**Fig. (1):**
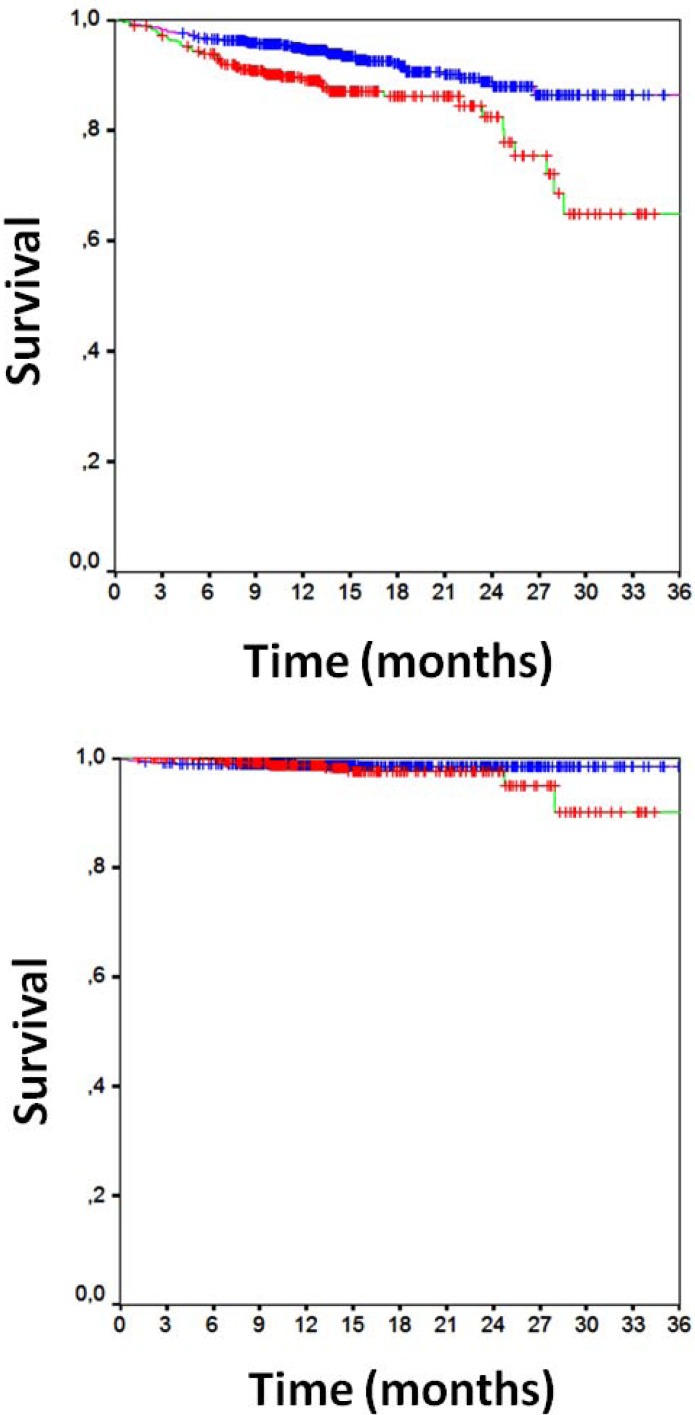
[**a**] Kaplan-Meier curve for survival free from major adverse cardiac events [direct stenting in blue, pre-dilation in red, p<0.001 at log-rank test]. [**b**] Kaplan-Meier curve for overall survival [direct stenting in blue, pre-dilation in red, p=0.265 at log-rank test].

**Fig. (2):**
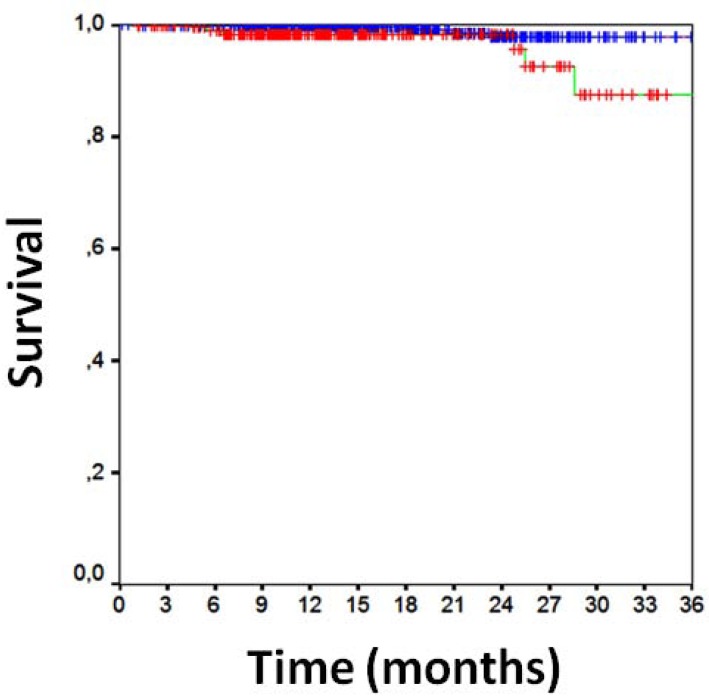
Kaplan-Meier curve for survival free from myocardial infarction [direct stenting in blue, pre-dilation in red, p=0.011 at log-rank test].

**Fig. (3):**
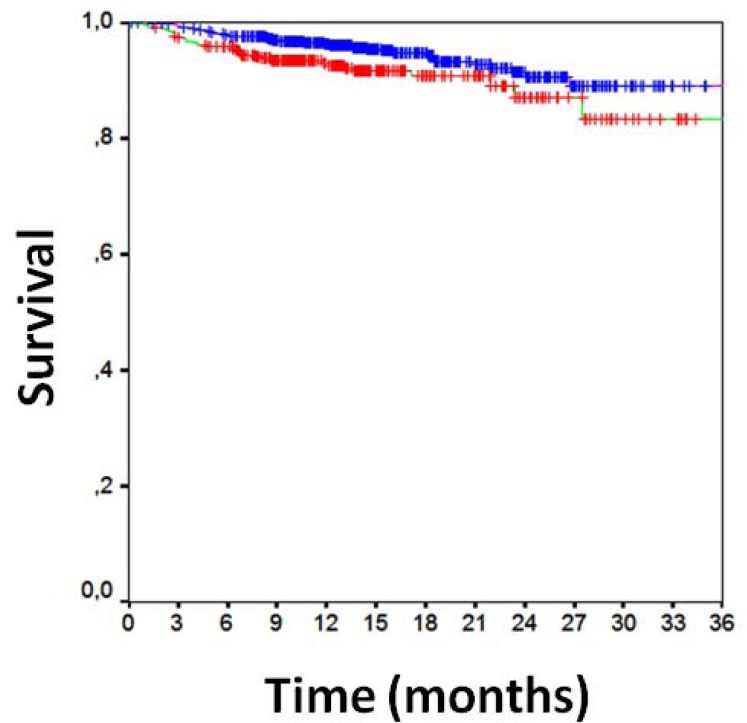
Kaplan-Meier curve for survival free from target vessel revascularization [direct stenting in blue, pre-dilation in red, p=0.024 at log-rank test].

**Fig. (4):**
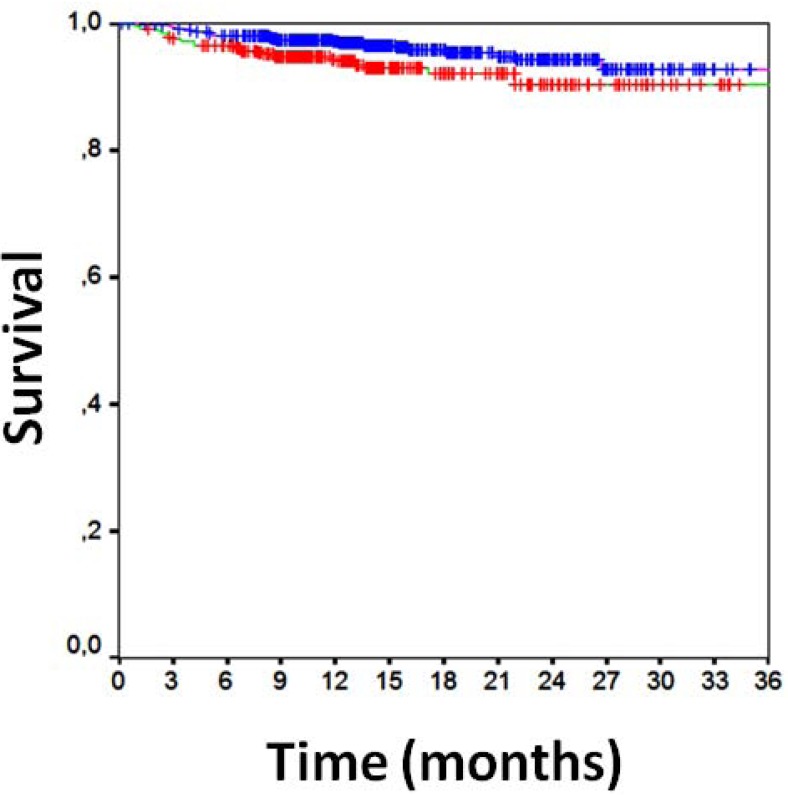
Kaplan-Meier curve for survival free from target lesion revascularization [direct stenting in blue, pre-dilation in red, p=0.031 at log-rank test].

**Fig. (5):**
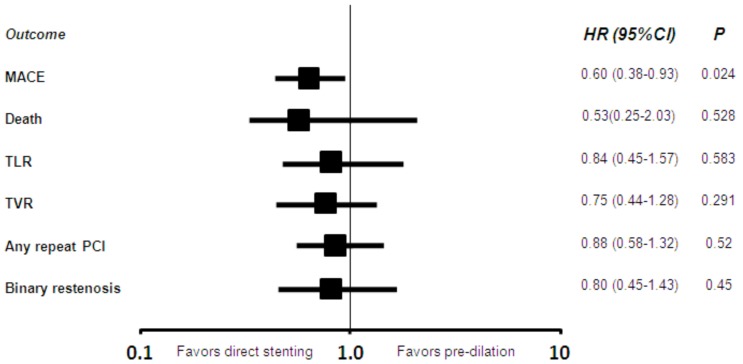
Propensity-adjusted hazard ratios [HR, with 95% confidence intervals [CI] and p values] for the risk of major adverse cardiac events [MACE], target lesion revascularization [TLR], target vessel revascularization [TVR], any repeat percutaneous coronary intervention [PCI] and binary restenosis.

**Fig. (6):**
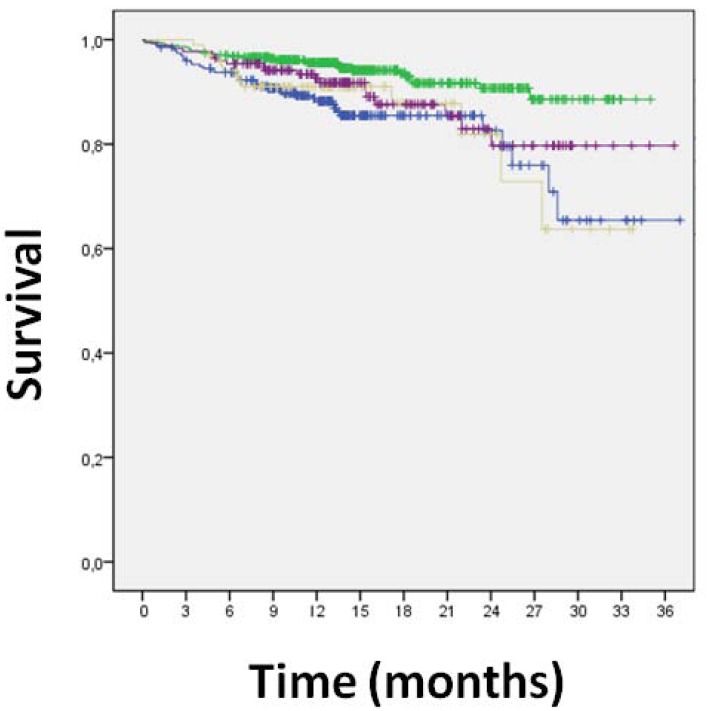
Kaplan-Meier curve for the interaction between direct stenting and diabetes mellitus [direct stenting in diabetics in purple, direct stenting in non-diabetics in green, pre-dilation in diabetics in brown, and pre-dilation in non-diabetics in blue].

Clinical follow-up data were available for all patients 16±7 months after PCI (Table **3**). Overall clinical results were remarkably favorable, with MACE in 10%, death in 2%, cardiac death in 1%, MI in 1%, TLR in 5%, TVR in 7%, any repeat PCI in 12%, CABG in 1%, and stroke in 1%. Focusing on the comparison of direct stenting versus stenting with pre-dilation, MACE had occurred in, respectively, 8% of those undergoing direct stenting versus 14% of those undergoing pre-dilation [p=0.001], with death in 1% versus 2% (p=0.380), cardiac death in 1% versus 2% (p=0.010), MI in 1% versus 2% (p=0.032), CABG in 0.2% versus 2% (p=0.012), TVR in 6% versus 9% (p=0.071), TLR in 6% versus 9% (p=0.071), and any repeat PCI in 11% versus 15% (p=0.065). Similar findings were obtained at unadjusted survival analyses for MACE (p<0.001, Fig. **1a**), death (p=0.265, Fig. **1b**), MI (p=0.011, Fig. **2**), TVR (p=0.024, Fig. **3**) and TLR (p=0.031, Fig. **4**). Even at multivariable analysis with propensity adjustment, direct stenting was associated with significantly fewer MACE in comparison to pre-dilation (hazard ratio 0.60 (0.38-0.93), p=0.024) and favorable trends for the other clinical outcomes (Table **4**, Fig. **5**) which were confirmed in diabetics as well as non-diabetics (Fig. **6**).

## DISCUSSION

The present observational study has the following implications: a) use of the novel thin-strut cobalt-chromium Skylor™ stent in this study based on such a single technology was associated with favorable results in unselected patients undergoing PCI for both elective and emergency indications; b) notwithstanding the inherent limitations of our observational study (i.e. selection bias) Skylor™ stent implantation and direct stenting technique appear beneficially synergic.

Despite the development of first-generation and subsequent generation DES,(14) BMS are still used in several thousand patients worldwide, especially in those at lower risk of restenosis and at higher risk of thrombosis or bleeding.( 6,8,15) Yet, the performance of currently available BMS is far from perfect, with restenosis being the most frequent and important complication. Improvements in BMS platforms have been mainly focused on developing devices with thinner struts, as strut thickness is strongly associated with restenosis,(7,9) and in compound alloys such as cobalt-chromium, which may provide greater flexibility and radiologic opacity, despite lower metal mass.(16) Indeed, BMS combining both thin-strut designs and cobalt-chromium alloys appear to improve early and long-term outcomes of PCI by, respectively, reducing the risk of side branch occlusion and preventing restenosis.(10,16-20) Additional benefits from the use of such devices can be envisioned when they are implanted with a direct stenting technique, as this approach is likely to maximize vessel scaffolding, minimize distal plaque embolization with ensuing microcirculatory injury, and geographical miss.(11-12, 21-22)

Our study thus sought to appraise the long-term outcomes associated with extensive use of the novel novel thin-strut cobalt-chromium Skylor™ stent in a large cohort of unselected patients with coronary artery disease, and simultaneously exploiting the revascularization strategy typical of our center, which is based on a routine adoption of direct stenting. Thus, we aimed to formally test the synergy between a direct implantation technique and the use of a highly flexible and deliverable thin strut cobalt chromium stent.

As many as 1020 patients were included, with 66% of them being treated with a direct stenting approach. After a 16-month follow-up, clinical results were highly favorable, with low rates of MACE (10%) and TLR (5%), which appear similar to those of of DES with higher late loss, such as first-generation zotarolimus-eluting stents [22]. Appraisal of the interaction between use of the Skylor™ stent and direct deployment technique showed that this revascularization strategy did not detrimentally impact on procedural success but was instead associated with significant clinical benefits at long-term follow-up. Thus, this work provides further evidence of the promising and beneficial role of new-generation BMS in routine clinical practice, and also strongly supports the default adoption of a direct stenting technique whenever such stents are employed.

Limitations of this work include the retrospective design, two-center enrolment, focus on a single device, and lack of formal appraisal for deliverability and flexibility. Indeed, a controlled study comparing Skylor to other stents would have been more informative in comparison to a single device registry, but such work was beyond our scope. In addition, the decision to perform direct stenting was not based on randomization. In addition, whenever direct stenting fails pre-dilation is required, and thus the actual group of patients receiving pre-dilation can best be viewed as composed by those with planned pre-dilation as well as bail-out pre-dilation. It is well known that incorrectly classifying these failing cases might lead to bias and spurious estimates [23-24]. We strived to minimized these confounding and biasing factors by avoiding losses to follow-up and extensive multivariable analysis with propensity score adjustment. confounding that may impact in this, as with any non-randomized trial, on comparisons and outcomes. We thus undertook extensive multivariable and propensity adjusted analyses to limit the extent of such confounding. However, unknown confounders could still impact and bias the study results and accordingly only a large randomized clinical trial could provide definitive evidence in favor of direct stenting when using the Skylor™ stent.

In conclusion, the MILES study suggests the presence of a beneficial synergy between direct coronary stenting technique and use of the novel thin-strut cobalt-chromium Skylor™ stent in real-world patients undergoing PCI.

## Figures and Tables

**Table 1.** Baseline Characteristics*

	Total (N=1020)	Direct stenting (N=672)	Stenting with pre-dilation (N=348)	P
Age	63.5±10.9	62.6±11.2	65.1±10.3	0.001
Male gender	821 (80.5)	538 (80.1)	283 (81.3)	0.630
Diabetes	286 (27.1)	183 (27.2)	93 (26.7)	0.863
Diabetes requiring insulin	55 (5.4)	37 (5.5)	18 (5.2)	0.823
Hypertension	669 (65.6)	431 (64.1)	239 (68.7)	0.139
Dyslipidemia	292 (28.6)	199 (29.6)	93 (26.7)	0.333
Hypercholesterolemia	136 (13.3)	79 (11.8)	57 (16.4)	0.039
Prior or current smoking	297 (29.1)	199 (29.6)	98 (28.2)	0.628
Renal failure	65 (6.4)	39 (5.8)	26 (7.5)	0.301
Prior myocardial infarction	228 (22.4)	143 (21.3)	85 (24.4)	0.253
Prior percutaneous coronary intervention	138 (13.5)	97 (14.4)	41 (11.8)	0.240
Prior coronary artery bypass grafting	76 (7.5)	52 (7.7)	24 (6.9)	0.628
Left ventricular ejection fraction <0.35	48 (4.7)	30 (4.5)	18 (5.2)	0.612
Acute coronary syndrome at admission	421 (41.3)	275 (40.9)	146 (42.0)	0.751
Acute or recent myocardial infarction	309 (30.3)	201 (29.9)	108 (31.0)	0.711
Multivessel disease	451 (44.2)	288 (42.9)	163 (46.8)	0.225

* reported as n (%) or mean±standard deviation

**Table 2.** Procedural and Lesion Characteristics*

	Total (N=1020)	Direct stenting (N=672)	Stenting with pre-dilation (N=348)	P value
**Procedures**	1099	704	395	-
**Treated lesions**	1.26±0.49	1.23±0.47	1.30±0.50	0.027
**Timing of procedure** **Elective** **Urgent** **Emergent**	886 (80.6) 211 (19.2) 2 (0.2)	577 (82.0) 126 (17.9) 1 (0.1)	309 (78.2) 85 (21.5) 1 (0.3)	0.311
**PCI of left main**	15 (1.3)	5 (0.7)	10 (2.5)	0.013
**PCI of left anterior descending**	371 (33.8)	232 (33.0)	139 (35.2)	0.452
**PCI of left circumflex**	334 (30.4)	215 (30.5)	119 (30.1)	0.886
**PCI of right coronary artery**	458 (41.7)	290 (41.2)	168 (42.5)	0.666
**PCI of other vessels**	30 (2.7)	22 (3.1)	8 (2.0)	0.283
**AHA/ACC type B2 or C lesion**	487 (44.3)	223 (31.7)	264 (66.8)	<0.001
**Total occlusion**	166 (15.1)	53 (7.5)	113 (28.6)	<0.001
**Ostial lesion**	24 (2.2)	16 (2.3)	8 (2.0)	0.788
**Bifurcation**	96 (8.7)	46 (6.5)	50 (12.7)	0.001
**Calcification**	90 (8.2)	46 (6.5)	44 (11.1)	0.008
**Tortuosity**	255 (23.2)	181 (25.7)	74 (18.7)	0.009
**Long lesion**	261 (23.7)	118 (16.8)	143 (36.2)	<0.001
**Small vessel disease**	438 (39.9)	238 (33.8)	200 (50.6)	<0.001
**Skylor™ stents**	1292	818	474	-
**Skylor™ stents per procedure**	1.18±0.45	1.16±0.44	1.20±0.46	0.253
**Skylor™ stent diameter**	3.03±0.43	3.09±0.43	2.93±0.39	<0.001
**Skylor™ stent length**	19.5±10.3	17.9±9.1	22.5±11.4	<0.001
**Post-dilation**	54 (4.9)	28 (4.0)	26 (6.6)	0.055
**Intravascular ultrasound**	1 (0.1)	1 (0.1)	0	1.0
**Procedural success**	1083 (98.5)	695 (98.7)	388 (98.2)	0.512
**Glycoprotein IIb/IIIa inhibitors**	1013 (92.2)	654 (92.9)	359 (90.9)	0.233
**Intra-aortic balloon pump**	0	0	0	1.0
**Dual antiplatelet therapy at discharge**	1016 (99.6)	640 (99.4)	376 (100)	0.126
**Duration of dual antiplatelet therapy (months)**	13.3±5.9	13.3±5.9	13.4±5.7	0.762

* reported as n (%) or mean±standard deviation; AHA/ACC=American College of Cardiology/American Heart Association; PCI=percutaneous coronary intervention

**Table 3.** Clinical Outcomes*

	Total (N=1020)	Direct stenting (N=672)	Stenting with pre-dilation (N=348)	P value
**Long-term (16-month) clinical outcomes**				
**Major adverse cardiac events**	101 (9.9)	49 (7.6)	52 (13.8)	0.001
**Death**	17 (1.7)	9 (1.4)	8 (2.1)	0.380
**Cardiac death**	10 (1.0)	4 (0.7)	6 (1.6)	0.010
**Myocardial infarction**	14 (1.4)	5 (0.8)	9 (2.4)	0.032
**Q-wave myocardial infarction**	2 (0.2)	1 (0.2)	1 (0.3)	1.0
**Target lesion revascularization**	51 (5.0)	26 (4.0)	25 (6.6)	0.065
**Target vessel revascularization**	68 (6.7)	36 (5.6)	32 (8.5)	0.071
**Any repeat percutaneous revascularization**	124 (12.2)	69 (10.7)	55 (14.6)	0.065
**Coronary artery bypass grafting**	7 (0.7)	1 (0.2)	6 (1.6)	0.012
**Stroke**	7 (0.7)	3 (0.5)	4 (1.1)	0.264
**Definite stent thrombosis**	3 (0.3)	1 (0.2)	2 (0.5)	0.284
** Angiographic outcomes**				
** Angiographic follow-up**	338 (33.1)	191 (29.7)	147 (39.1)	0.002
**Timing of angiographic follow-up**	9.7±8.4	10.5±8.4	8.7±8.2	0.056
**Binary angiographic restenosis**	66 (6.5)	29 (4.5)	37 (9.8)	<0.001

* reported as n (%)

**Table 4.** Multivariable Analysis*

	Adjusted hazard ratio of direct stenting†	95% confidence interval	P value
**Major adverse cardiac events**	0.599	0.384-0.934	0.024
**Death**	0.528	0.250-2.034	0.528
**Target lesion revascularization**	0.840	0.452-1.563	0.583
**Target vessel revascularization**	0.748	0.435-1.283	0.291
**Any repeat percutaneous revascularization**	0.875	0.582-1.316	0.521
**Binary angiographic restenosis**	0.799	0.446-1.432	0.451

* Adjusting for age, diabetes mellitus, dyslipidemia, renal failure, acute or recent myocardial infarction, depressed left ventricular ejection fraction, multivessel disease, number of treated lesions, American College of Cardiology/American Heart Association type B2 or C lesion, left main, total occlusion, bifurcation, calcification, tortuosity, long lesion, small vessel, stent diameter, stent length, and post-dilation, with an enter model

† Similar findings obtained after fitting in the regression model a parsimonious propensity score with area under the curve=0.786 and p for association to direct stenting<0.001
